# Estimating short and longer-term exposure of domestic cats to dietary iodine fluctuation

**DOI:** 10.1038/s41598-022-13139-8

**Published:** 2022-05-28

**Authors:** R. Alborough, P. A. Graham, D. S. Gardner

**Affiliations:** School of Veterinary Medicine and Science, University of Nottingham, Sutton Bonington Campus, Nottingham, LE12 5RD UK

**Keywords:** Animal physiology, Kidney, Experimental models of disease

## Abstract

Hyperthyroidism is a common endocrinopathy of domestic felines. In humans, toxic nodular goitre is pathophysiologically similar to feline hyperthyroidism and can be caused by chronically low or fluctuating dietary iodine intake. The aetiopathogenesis of feline hyperthyroidism is not known, but chronically low or fluctuating dietary iodine intake is likely common. This study assessed habitual iodine intake in domestic cats by: (1) conducting a dietary survey involving 361 owners of 549 cats, (2) analysing iodine content of 119 cat feeds, 38 urine and 64 hair samples and (3) assessing variation in iodine content of eight cat feeds over 4–6 different batches. Owners varied their cats feed regularly, usually on a day-to-day basis and often between wet and dry feeds with differing flavours. The majority (78%; 93 of 119) feeds for cats were within the guideline range for iodine. Of the 22% (n = 26 feeds) that were not compliant, the majority (n = 23) were below the nutritional minimum value with most (n = 16) being dry kibble. Iodine content of feeds did not vary considerably between types of feed or feed packaging, but variation between different batches of the same feed was 14–31%. Hence, urine iodine in cats also varied markedly. Cats being treated for hyperthyroidism had lower hair iodine. In conclusion, a survey assessing how domestic cats are fed, together with an analysis of commercial cat feeds suggests that domestic cats are likely to experience chronically low or fluctuating dietary iodine intake. The latter is supported by wide variation in urine iodine content.

## Introduction

Hyperthyroidism is an endocrinopathy associated with an overactive thyroid gland, unresponsive to negative feedback, resulting in chronically elevated thyroid hormone production. Hyperthyroidism is the most common endocrinopathy of domestic cats^1,2^. In domestic dogs, hyperthyroidism is relatively rare^3^. The prevalence in domestic cats, worldwide, has increased rapidly since the first definitive diagnosis in 1979^4,5^. Whilst increased diagnosis and greater availability of better assays may have contributed to this increase^5,6^, many other risk factors have been proposed, including; increased age^7,8^, increased time spent indoors^9^, breed-specific predisposition^8–12^, use of cat litter^8,11^ or dietary factors such as a lack of supplementation in commercial foods^13^, increased feeding of canned feeds^6,8,9,11^ or increased consumption of fish^14^.

There are striking similarities between feline hyperthyroidism and human toxic nodular goitre (TNG)^15^. TNG is a form of hyperthyroidism that occurs especially in elderly human patients living in iodine-deficient areas^16^. Iodine (as iodide in its ionic state in the body) is the fundamental element required to manufacture thyroid hormone^17^. The higher incidence of TNG in iodine-deficient areas is thought to be primarily due to fluctuations in dietary iodine intake^16,18^, but an alternative aetiopathogenesis has also been proposed—bioaccumulation of environmental contaminants in the thyroid gland itself^19^. Nutritionally, high circulatory iodide (e.g. after high dietary iodine intake) inhibits sensitivity of the thyroid gland to thyroid stimulating hormone (TSH), amongst other effects, to ultimately reduce thyroid hormone secretion^20,21^. Low circulatory iodide leads to an upregulation of cellular mechanisms in the thyroid gland to increase incorporation of any remaining iodide into production of increased thyroid hormone^18^. In essence, follicular cells in the thyroid gland form nodules that become autonomous, growing and functioning independently from TSH control^18,22^. Problems arise if iodine supply suddenly increases; prior adaptation to low iodide supply in the face of an acute increase in iodine, leads to the thyroid gland being unable to acutely down-regulate TSH production; thyroid hormone production continues, secretion increases and hyperthyroidism may ensue^16^.

In domestic cats with hyperthyroidism, the pathophysiology is similar to TNG; the thyroid gland becomes nodular with thyroid follicular cells gaining independence from TSH control^15^. However, unlike human TNG, the pathogenesis (i.e. fluctuations in dietary iodine intake) is largely unknown, but has been speculated^13^. Higher levels of environmental contaminants have been found in the thyroid glands of cats with hyperthyroidism^23,24^. Nutritionally, circulating thyroid hormone concentrations may be altered by change in dietary iodine^25^ and urinary iodine content mirrors dietary intake^25–29^. Paetau-Robinson, et al.^27^ investigated the effect of feeding a limited iodine diet to healthy, young to middle-aged adult cats for two years, and found that serum free thyroxine (fT4), total T4, free triiodothyronine (fT3), total T3 and TSH concentrations, on average, were maintained within the normal reference ranges. In the domestic cat, the potential for fluctuation in dietary iodine to interact with nodules in the thyroid to result in high T3/T4 output is not known. Nevertheless, given the similarities between TNG and feline hyperthyroidism, it would appear prudent to consider consistency in dietary iodine provision for cats, something that has not been investigated to date.

In previous studies, complete feed for domestic cats was shown to have highly variable iodine content, with some of the feeds analysed having above-recommended iodine content^26,30,31^. Therefore, it appears plausible that domestic cats may experience fluctuations in their iodine intake over time. To date, in the UK, no study has assessed iodine content of commercially available pet foods, nor asked owners what they routinely feed their cats. Feeding canned commercial pet foods to cats^6,8,9,11^ or fish-based pet foods^8,14^ has been associated with an increased risk of developing feline hyperthyroidism. Fish, in general, have relatively high iodine content^32^ and are fed to cats more often than to dogs^33^.

In this observational study, we have first asked, by survey, what owners routinely feed their cats to ascertain variability of feed intake according to main flavour and type of food. Secondly, we have measured the iodine content of a range of cat feeds typically bought, and fed to, cats in the UK. Third, we have assessed how variable feed iodine content is across multiple batches of the same feeds with relatively high or low iodine content. Finally, we measured iodine content in the urine of cats as a potential marker of acute variability in dietary iodine and, for the first time, measured iodine content in hair as a speculative measure of longer-term iodine exposure. For mineral analyses of feed, reference has been made to EU guidelines for nutritional minimum and maximum levels of iodine in complete diets^34^.

## Materials and methods

### Ethics and study approval

This study was approved by the University of Nottingham School of Veterinary Medicine and Science Research Ethics Committee (REC 2074 170721) and all methods were carried out in accordance with the relevant guidelines and regulations for good laboratory practice. Informed consent was obtained from pet owners to collect and use hair or urine from their pet for research. Urine samples were collected from the bladder at necropsy by board-certified pathologists.

### Collection of hair and urine samples

Hair from healthy cats (n = 38) not currently being treated for, and without a known history or signs of, hyperthyroidism was collected by their owners through grooming. Hair from cats actively being treated for hyperthyroidism (n = 26) was obtained indirectly by using clippings during routine blood sampling. Clinical and laboratory findings consistent with a diagnosis of hyperthyroidism as determined by primary care veterinary surgeons and re-confirmed on admission to the referral veterinary hospital prior to radioactive iodine therapy constituted the diagnosis of hyperthyroid cats for the purposes of this study. Information, such as age and breed, was collected for all of the healthy control and hyperthyroid cats. Median (interquartile range) ages were 78 (37–96.75) and 146 (131–163.5) months for healthy control and hyperthyroid cats, respectively. The majority of control (n = 28 of 38; 73.7%) and hyperthyroid cats (n = 19 of 26; 73.0%) were domestic short-haired (DSH). Other breeds included domestic longhair, Ragdoll, British shorthair, Siamese and Maine Coon-cross. The breed was unknown for n = 4 cats. Urine (1–2 ml) was obtained directly from bladders of previously euthanised (under auspices of Veterinary Surgeons Act) domestic cats (n = 43) submitted to The University of Nottingham Veterinary Pathology Service for necropsy. For reference, and for comparison of the iodine content of canine feed (Figure S1a) and canine urine from healthy dogs, we used a population of domestic dogs (n = 52; Figure S1b) similarly submitted to pathology as for the feline cohort. Dogs share the same environment as domestic cats and are exposed to potentially similar variation in dietary iodine through consumption of commercial foods bought in the UK, albeit with differing nutrient requirements.

### Selection of pet foods

A range of commercially available, wet (n = 56) and dry (n = 63), cat foods that had previously been analysed for mineral content were selected for determination of iodine content^35^. These foods were purchased from local supermarkets and pet shops and comprised a range of familiar brands sold in the UK. All (n = 119) of the foods were labelled as ‘complete’ as opposed to ‘complementary’. The foods were packaged in a range of sealed tins, cans, pouches or bags. Foods of a variety of main flavours, determined by the first ingredient listed on the packaging as comprising the highest proportion of the diet, were included. The majority of the foods analysed in the current study were intended for consumption by adult cats or dogs, although some were designed for young or senior animals. In addition, eight of the complete wet foods for adult cats (Foods 1 to 4 with relatively low iodine content and Foods 5 to 8 with higher iodine content, as determined by initial iodine analysis) were re-purchased over time to obtain between four and six different batches (according to Batch ID on the label) for each of these feeds.

### Preparation of raw samples for iodine analysis

#### Hair samples

were stored in sealed plastic bags in the dark at room temperature until processing; a method of storage reported to avoid compositional change in hair over time^36^. Gloves were worn throughout preparation of the hair samples to prevent contamination. Each hair sample was washed, as previously described^36,37^. In brief, hair samples (~ 0.5 to 1.0 g) were immersed in 3:1 (v/v) ethyl-ether:acetone, stirred for 5 min to remove any sebaceous film coating on the hair and dried. Subsequently, hair was placed in 5% ethylenediaminetetraacetic acid (EDTA) for 1 h to chelate any external chemical elements on the hair surface. Finally, each sample was rinsed three times with deionised milli-q water for 15 min. Washed hair samples were transferred to labelled 250 ml solvent-resistant containers (Sarstedt, UK) and thoroughly dried at 50 °C for 48 h.

#### Urine samples

were stored at −80 °C until processing. Thawed samples were centrifuged for 5 min at 3222 *rcf* (2000*g*), and 500 µl supernatant added to a 14 ml (105 mm × 16.8 mm) polypropylene tube (Sarstedt, Leicester, UK), diluted 1 in 20 by addition of 9.5 ml of 1% tetramethylammonium hydroxide (TMAH).

#### Pet feed samples

representative quantities (100–200 g) or the whole sample (e.g. from pouches, tins, trays), were freeze-dried (−55 °C; 48–72 h; ScanVac CoolSafe 55-9 freeze-dryer), ground to a homogenous powder and stored in sealed containers in the dark, until later analysis.

### TMAH digestion of prepared samples (excluding urine)

Duplicate 100–200 mg hair/feed were weighed into 50 ml inert polytetrafluoroethylene (PTFE)-tetrafluoroethylene-modified (TFM) lined vessels and 5.0 ml, 10% TMAH added. After incubation for 1 h, vessels were transferred to a 24HVT50 microwave (Multiwave PRO, Anton Parr, Austria) to accelerate digestion by 45 min heating. For every 11 samples digested in duplicate, two digestion vessels were left empty (blank; with no hair or food sample) with only 5.0 ml of 10% TMAH added to control for any within-run contamination. Digested samples (5.0 ml) were decanted into 50 ml centrifuge tubes and 45 ml deionised milli-q water added (1:10 dilution, final 1% TMAH matrix). All samples were then centrifuged for 30 min at 2000*g*. An aliquot (5–10 ml) of each sample was used for analysis of iodine by inductively coupled plasma mass spectrometry (ICP-MS).

### Iodine analysis by inductively coupled plasma mass spectrometry (ICP-MS)

An ICP-MS iCAP-Q, (Thermo-Fisher Scientific, Loughborough, UK) was used for iodine determination in all prepared samples, blanks and a certified reference material (CRM). The CRM used was SERO210705 Seronorm Trace Elements Urine L-2 (LGC Standards), with a known iodine content of 297 μg/L, which was added in duplicate per ≤ 60 samples and blanks. Diluted samples were introduced via a covered autosampler (Cetac ASX-520) through a 1317090 nebulizer (ESI; Thermo-Fisher Scientific, Loughborough, UK). ICP-MS used a ‘Flatopole collision cell’, which was charged with helium gas. The collision cell was upstream of the analytical quadrupole to reduce polyatomic interference. An internal standard of 5 ppb rhenium in 1% TMAH was introduced to the sample stream via a T-piece. External calibration standards were in the range of 0–100 μg/L (ppb). Sample processing was undertaken using ‘Qtegra software’ (Thermo-Fisher Scientific, Loughborough, UK). Final iodine content in each sample is presented after correction for any iodine in blanks and batch recovery against the CRM (n = 14 independent runs, 89% recovery, 6% coefficient of variation [CV]).

### Reproducibility of iodine measurements

Intra-assay CV was determined by analysing n = 3 independent biological replicate hair or feed samples each with n = 6–8 technical replicates on the same ICP run. Inter-assay CV was determined by analysing n = 12 different hair or feed samples in duplicate, over two separate ICP runs. Intra- and inter-assay variation for iodine in hair were both 11%. Intra- and inter-assay variation for iodine in pet feed were 3% and 5%, respectively.

### Human hair iodine

Since, to our knowledge, iodine content of cat hair has not been previously measured or published, no acceptable ‘normal range’ existed. Therefore, to validate the methods in our hands we measured iodine in human hair for comparison against values previously reported^38^. Hence, n = 10 healthy humans without known thyroid disease recruited via a process of informed consent (REC: 252-1802, University of Nottingham Faculty of Medicine Research Ethics Committee) were asked to collect hair either by brushing over several days, or excess after cutting. Samples were processed as described above and data are shown for reference in Fig. S1c.

### Urine iodine:creatinine content

Urine creatinine (µmol/L) was measured in each neat, urine sample (100–200 μl) using an automated assay, based on the enzymatic reaction of creatinine with picric acid, producing a colorimetric product (RX-Imola; Randox). The assay and standards were used according to the manufacturer’s instructions (Randox Laboratories Limited, 2018). Human-based urine (Assayed Urine Control Level 3, AU2353) with known creatinine content was used as the quality control (QCs) to correct for intra- and inter-assay variability. Concentrations of creatinine measured in urine samples were converted from μmol/L to g/L, using the molecular weight of creatinine (88.42 g/mol) in order to express urine iodine (µgs) on a mass corrected basis (μg iodine per g creatinine).

### Survey of cat feeding habits

To determine whether cats are commonly fed the same diet or whether diets are altered on a regular basis, a survey was created using Jisc Online Surveys. The survey was approved by the Clinical Ethical Review panel at the University of Nottingham School of Veterinary Medicine and Science (UoN-SVMS) and underwent a pilot study (n = 4 respondents) prior to distribution via social media and cat owner forums, and by email, among staff and students at UoN-SVMS. Respondents could answer the survey for up to three cats that they owned. If they owned more than three cats, they could access the survey again to complete it for any additional cats. Closed-ended questions were included in the survey to collect information about the identity of the cat, such as breed, sex and neutered-status, date of birth and health status. Free-text answer boxes were provided to obtain extra information about any specific diseases or signs of illness affecting the cats in this study, as well as the specific veterinary-prescribed diet where these were fed. A question regarding how many cats lived within the household was also included. A series of multiple-choice questions were used to determine the proportions of different types of food (commercial diet: dry, wet [canned, pouches, foil trays, other packaging], flavours [fish, red meat, white meat]; non-commercial diet: home-prepared diet, leftovers, table-scraps, other]) that each cat was fed and the frequencies of dietary changes (by type [wet vs. dry], flavour, brand) and provision of treats. Free-text answer boxes were provided to obtain additional details of the types of treats offered to cats. Questions regarding the amount of time spent outside on average within a 24-h period, the frequency of prey-consumption and the prey species consumed were also included. At the end of the survey, respondents could select whether they lived in or outside of the UK. A notes section was included at the end of the survey for any additional comments that survey respondents wanted to leave.

### Statistical analysis

Data for the iodine content in control versus hyperthyroid cats’ hair were compared using the Mann–Whitney U test, due to the non-normal distribution of the data. Cat and dog corrected urine iodine content were analysed using linear mixed models after normalisation of the distribution by log-transformation of the raw data. Statistical significance was accepted at P < 0.05. Data are reported as median (IQR: 1st to 3rd quartiles). Pet food iodine content was also log-transformed prior to analysis (by ANOVA) to normalise the distribution of the data. Where marked unequal group sizes were present, such as analysing iodine content in different packaging types (plastic trays, foil trays, pouches and cans), then mixed-effect models were used to estimate significant differences in an unbiased way. Statistical significance was accepted at P < 0.05 and all analyses used GenStat v21 (VSNi Ltd, Rothamsted, UK).

Survey responses for cats within the UK and cats outside of the UK were grouped separately for an initial statistical analysis using linear mixed models to test whether geographical location had an effect on feeding habits. Since country had no effect, these groups were combined for all subsequent analyses. Respondent ID was applied in a random model to account for cats within each group that may have a shared owner and thus, potentially more similar feeding habits, compared to cats from single-cat households. Statistical significance was accepted at P < 0.05. Pearson chi-square test was used to determine any effects of multi-cat versus single cat households on the feeding habits data. Linn’s coefficient of concordance was used to evaluate the correlation between the proportions of wet food fed to two cats within the same household. Kendall’s coefficient of concordance, adjusted for ties, was used to assess the degree of agreement between the proportions of wet food fed to three different cats residing within the same household. For these multi-cat analyses, there were n = 136 owners that had two cats and n = 33 of these owners had a third cat. There were four cats in n = 5 households, a fifth cat in n = 4 of these households, a sixth cat in n = 3 households and eight cats in n = 1 of these. Where there were four or more cats within a household, only responses for up to the first three cats, for which the owner completed the survey, were formally analysed due to insufficient numbers of owners with four or more cats. Statistical significance was accepted at P < 0.05. To check that the percentage of total cats for which each multiple-choice answer was selected was not skewed by potentially identical feeding practices for multiple cats within a single household, data from only the first cat of each owner was used and the percentages of these cats that represented each multiple-choice answer were compared to the percentages obtained when all cats were included.

### Power calculation and sample size

An interim power calculation was conducted after one year of hair sample collection when iodine content in n = 12 hyperthyroid and n = 38 euthyroid cats had been measured. In order to detect an effect size of ~ 50% (median difference in average of each population) at a two-sided significance level of 0.05 and a power of 80%, each study population would require n = 32 samples.

## Results

### What are cats in the UK fed and how variable could iodine intake be?

*Descriptive statistics of our respondents:* The survey was completed by a total of 361 owners of 549 cats, of which n = 421 were based in the UK. The majority of international respondents were either from North America (79 of 128 cats; 62%) or Europe (n = 22 cats). There was no overall difference (*P* = 0.70) in responses according to geographical region and therefore these data were combined to assess overall feeding habits of cats. The most represented breed of cats was domestic short- (n = 381; 69.4%) or long-haired (n = 57; 10.4%). All other cats were either pure- or cross breeds. The dataset was balanced for male and female cats (n = 256 and 274, respectively) and the overwhelming majority were neutered (spayed female: n = 263 of 530, 49.6%; entire female: n = 11, 2.08%; castrated male: n = 246, 46.4%; entire male: n = 10, 1.89%). The average age of cats was 7.28 (± 4.87) mean (± 1S.D.). Eighty-four (15%) cats were reported to be ‘unhealthy’ (i.e. had a disease recognised by their vet, and/or signs of illness), and this was commonly reported as being hyperthyroidism (n = 16; 19.0%), chronic kidney disease (CKD; n = 13; 15.5%), arthritis (n = 12; 14.3%), inflammatory bowel disease (n = 7; 8.33%) or diabetes (n = 6; 7.14%). The hyperthyroid cats were 14.5 ± 3.1 yrs of age, mostly female (n = 12/16), average weight, 3.67 ± 1.21 kgs and, in common with the majority of other cats in the survey, were nearly all fed commercial dry food. There were 60 other reports of 39 different conditions. Veterinary prescribed diets were fed to 77 (14%) cats, which included 32 cats with reported ill-health and 45 healthy cats. The survey was completed for 222 cats that did not live with another cat and 327 cats that lived in multi-cat households (n = 138 households). Most (n = 104; 75.4%) owners that completed the survey for multiple cats had only two cats. However, 27 (19.6%) owners had three cats, two (1.4%) had six cats, and one owner (0.7%) had eight cats.

*Are cats in single or multi-cat households fed differently?* The proportion of wet food that constituted a commercial diet fed to cats was not different between cats in single versus multi-cat households (χ^2^ [4 d.f., n = 535] = 2.89, *P* = 0.57; Table S1). For cats in multi-cat households, there was no significant difference in the proportions of wet food fed to cat 2 or cat 3, versus cat 1 (χ^2^ [8 d.f., n = 33] = 2.4, *P* = 0.96). Indeed, Linn’s coefficient of concordance showed that, in households with at least two cats, the proportion of wet food fed to cat 2 was highly concordant with that fed to cat 1 (Cb 0.89, n = 136 households, n = 272 cats; Table S1). In households with three or more cats, cats 2 and 3 were highly likely to be fed the same proportion of wet food as cat 1 in that household (Kendall’s coefficient of concordance, adjusted for ties: 0.72; χ^2^ [32 d.f., n = 33] = 69, *P* =  < 0.001; Table S1). For the five owners with ≥ 4 cats the responses were 100% concordant. Similar to responses by country, there was no effect on responses from single or multi-cat owners (Table S2) and thus, for further data presentation and analyses, we retained responses for all cats and the results of this survey are expressed as ‘number of cats’ rather than ‘number of owners’.

#### What type of food is commonly fed?

**Figure 1:**
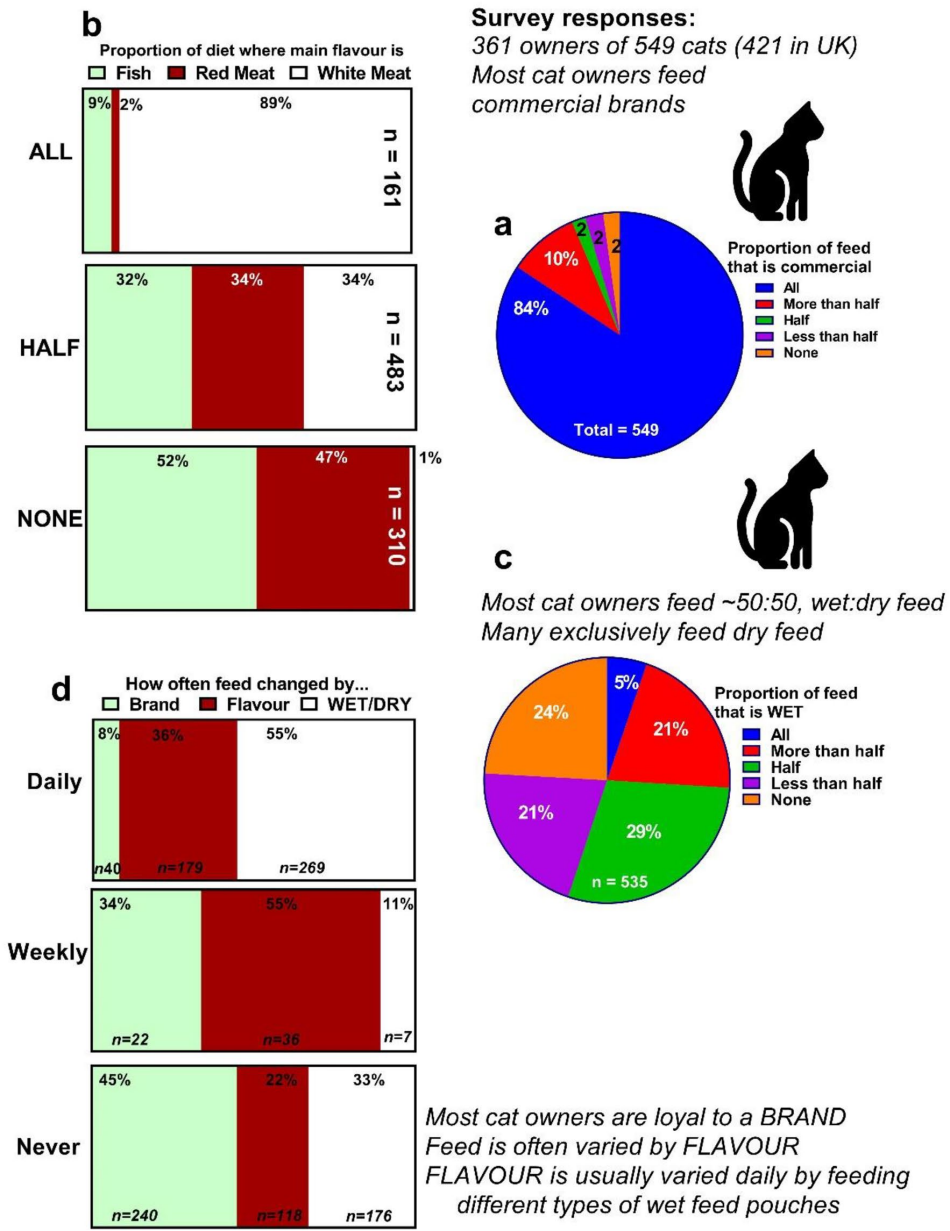
An international survey of cat owners regarding their feeding habits. Survey responses from n = 361 cat owners of n = 549 cats about their cats’ feeding habits. (**a)** Proportion of feed that is ‘commercially-available’ cat food, rather than home-made. (**b)** Proportion of feed according to main flavour given to cats; (**c)** proportion of the cat’s commercial diet fed as wet, as opposed to dry, feed; (**d)** proportion of owners changing the brand, flavour or type (e.g. wet to dry) of feed given to their cats. The survey of cat feeding habits was created using Jisc Online Surveys. The numbers indicate the number of cats, rather than number of owners, for which the particular response option was selected. Any variation from expected proportions were analysed using Pearson chi-square, as indicated in text of results. Cartoon image of a cat obtained from Microsoft, CC-BY-4.0 licence.

The majority (463 of 549; 84.3%) of cats were fed *only* commercially available cat food (Fig. 1a). A further 9.3% of respondents reported feeding their cats commercial feed ≥ 50% of the time. Very few owners reported feeding less than half, or no, commercial feed (2.4% and 2.2%, respectively). For those respondents where commercial cat food did not comprise the entirety of the cats’ diet, the remainder was a mixture of home-prepared diets (30 of 86 [34.9%] cats), table scraps (31 of 86 [36.0%] cats), leftovers (29 of 86 [33.7%] cats), or other items such as human foods (21 of 86 [24.4%] cats) or prey items (16 of 86 [18.6%] cats). When feeding commercial diets, few owners exclusively fed wet foods (n = 28 of 535, (5.2%)), whereas many more did so for dry foods (129 of 535; 24.1% cats). A large proportion of owners tended to feed both wet and dry feeds in combination, with either wet (156 of 535; 29% cats) or dry (111 of 535; 20.7% cats) making up the greater proportion (Fig. 1b). Hence, what owners fed cats in terms of wet/dry was significantly different (χ^2^ [4 d.f., n = 535] = 85.59, *P* ≤ 0.001). If wet food in only one packaging type was fed to cats, this would most likely be as pouches (210 of 394; 53% cats), followed by cans (71 of 394; 18% cats). Indeed, only 88 cats (22.3%) did not receive any wet food in pouches.

#### What flavours are commonly fed?

Meat-flavoured foods were fed more commonly than fish-flavoured foods, with the latter making up either none (n = 161; 30%) or less than half to half of the total diet (n = 323, 60%). Nevertheless, 14 of 541 (2.6%) respondents reported feeding their cats exclusively fish (Fig. 1c). The types of meat-flavoured food making up the greater majority of the diet given to cats tended to be white meat (e.g. chicken, turkey; 286 of 486 [59%]) followed by red meat or a combination of the two; a third (164 of 486; 33.7%) of cats were said to receive half white and half red meat as their main flavour of feed.

#### How often are feeds varied?

Only 83 of 524 (15.8%) cats were reported to never experience dietary changes of any form. Nearly half (240 of 524 [45.8%]), however, never experienced a change in the *brand* of pet food. In those where the brand was reported to change, this was only on a less than monthly basis in most cases (141 [26.9%]). Very few altered the brand fed on a regular basis e.g. daily, 7.6%; weekly, 4.2%; monthly, 8.4% of cats (Fig. 1d). The *type* of food (wet or dry) that a cat receives was never changed for 175 of 524 (33.4%) cats. 266 of 524 (50.8%) cats were given both wet and dry food each day. In contrast to the brand and type of feed, the flavour of pet food was routinely varied on a daily (179 of 524, 34.2%) or weekly (96 of 524, 18.3%) basis. The flavour of food was never changed for 118 (22.5%) cats. Most cats in our survey were either fed twice a day (n = 188; 34.7%) or had dry food freely available (i.e. ‘ad lib’ (n = 189; 34.9% cats)). Ad lib feeding was as common as feeding twice a day. Very few cats (n = 17; 3.1%) were fed only once per day. Most cats (239 of 524, 44.9%) ate all the food given to them ‘most of the time’.

### Iodine content of cat feeds

**Figure 2:**
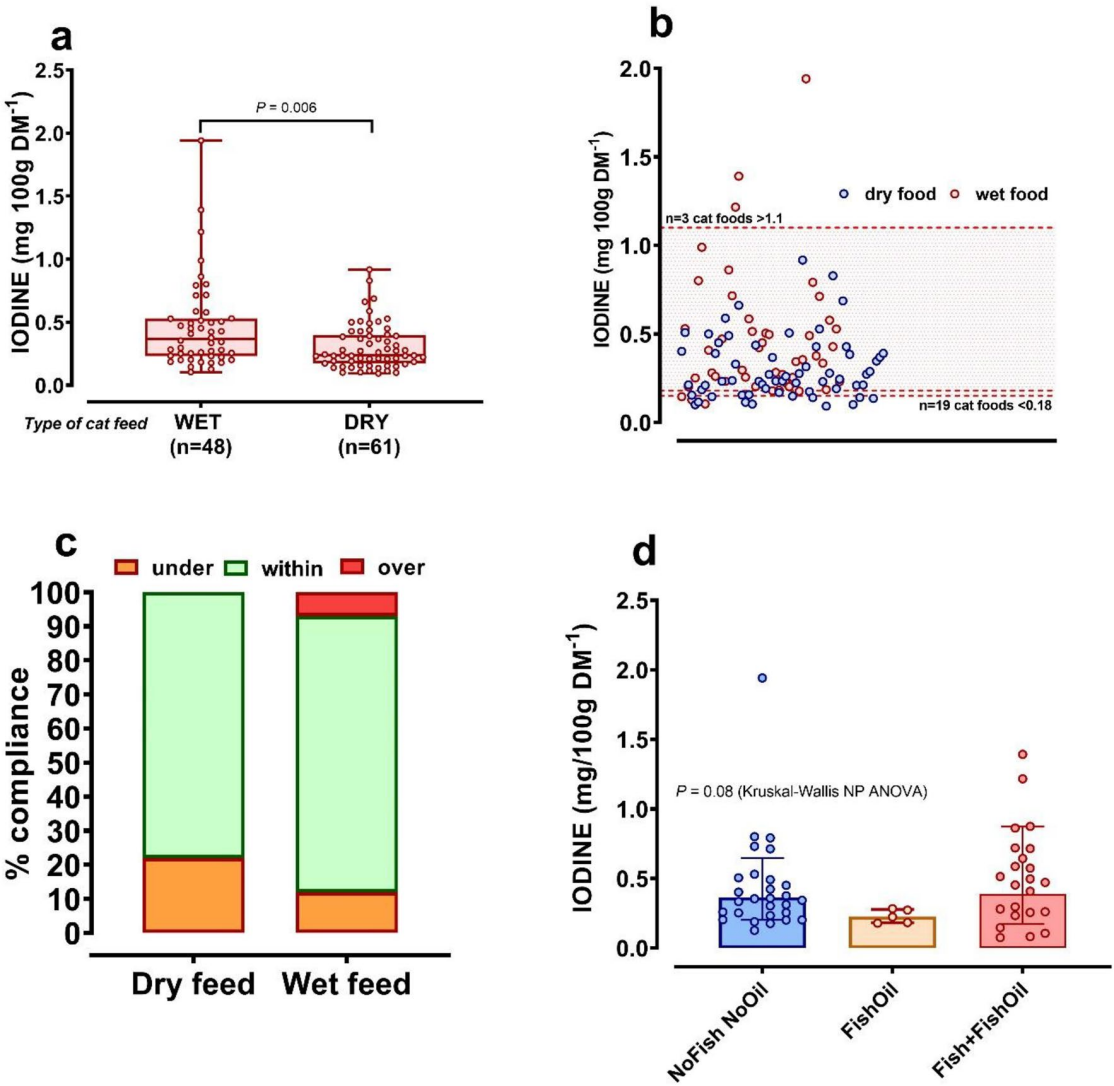
Iodine content of complete, wet and dry, dog and cat foods. Freeze-dried wet and dry pet foods were homogenised and duplicate samples (100–200 mg) were digested with TMAH for subsequent analysis of their iodine content by ICP-MS. **(a)** Iodine content of wet and dry cat foods where each food is represented by an individual dot, boxes show upper to lower quartiles with line at median, whiskers from min to max, data analysed by Mann–Whitney U-test. **(b)** Assessment of compliance of individual wet (red dots) and dry (blue dots) cat foods with FEDIAF recommendations for the nutritional maximum (1.1 mg/100 g DM) and minimum (0.18 [kitten], 0.17 [cat], mg/100 g DM^−1^) iodine content of pet foods, represented by dotted lines. **(c)** Data are proportions of cat feeds analysed under, within-range or over FEDIAF guidelines. **(d)** Iodine content of wet foods grouped according to whether the feed contained no fish and no fish-oil (NoFishNoOil), with only fishoil added (FishOil) or with both Fish and fish oil (Fish + FishOil). Dots represent individual foods, boxes show the geometric mean and whiskers show the geometric standard deviation of the data. Data were analysed by non-parametric analysis of variance (NP ANOVA). Statistical significance was accepted at P < 0.05.

Wet foods had higher iodine content (mean, 0.44 mg iodine/100 g DM^−1^) and a wider range of iodine (0.07–1.94) than dry foods 0.30 (0.09–0.91), respectively; *P* = 0.006 (Fig. 2a). Overall, 78.1% (93/119) cat foods analysed complied with both the minimum and maximum level of iodine in feed as recommended by FEDIAF (Fig. 2b,c). Of the n = 26 feeds that did not comply, n = 3 of 56 wet feeds analysed were above recommended guidelines, whereas n = 7 wet (of 56) and n = 16 dry (of 63 dry feeds analysed) were below recommended guidelines, which were nutritional minimum for adult cat foods, 0.17 mg/100 g DM^−1^, minimum for growth and reproduction, 0.18 (kitten) mg/100 g DM^−1^ and EU legal maximum: 1.1 mg/100 g DM^−1 34^. There were few consistent effects noted according to packaging type or food constituent (e.g. branded as ‘fish’ or not; Fig. 2d). For example, pet feed with fish as the main flavour had no greater iodine content than pet feed without fish (with fish, 0.49 ± 0.44; without fish, 0.42 ± 0.47 mg/100 g DM; *P* = 0.37).

### Batch-to-batch variation in feed iodine content

**Figure 3:**
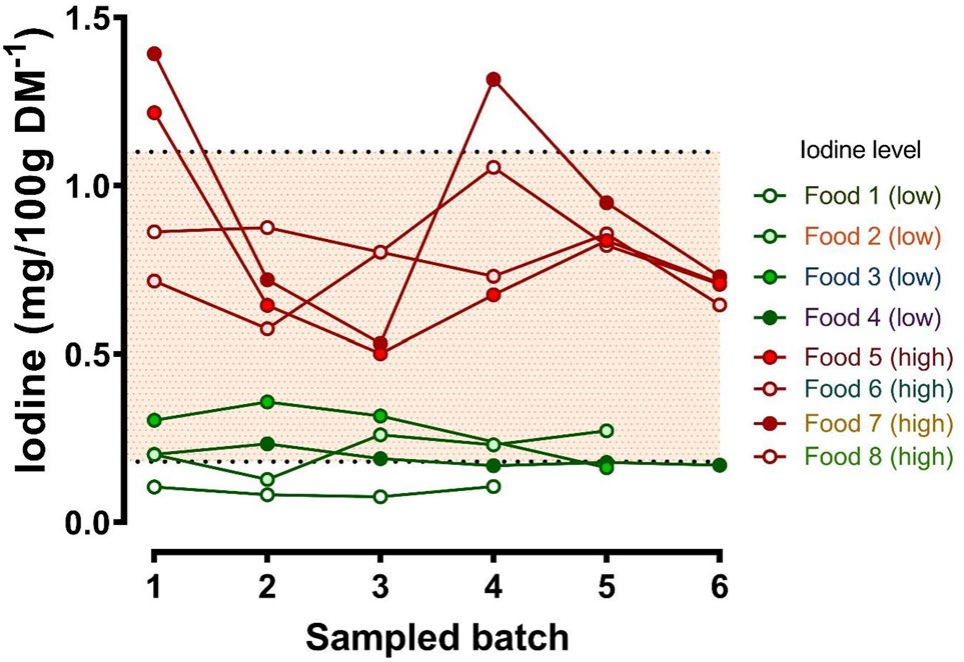
Batch-to-batch variation in iodine content of complete wet feed for pet cats. Iodine was measured by ICP-MS in duplicate samples (100–200 mg) of the freeze-dried, homogenised, TMAH-digested foods and corrected to the iodine concentration measured in duplicate TMAH-digested blank samples and certified reference material (CRM; SERO210705 Seronorm Trace Elements Urine L-2; LGC Standards). Dashed lines show the legal maximum and recommended minimum level of iodine that complete foods for adult cats should contain, according to FEDIAF.

Analysis of the iodine content of the same brand and type of feed on multiple occasions, with either known low (feeds 1–4; Fig. 3) or higher iodine content (feeds 5–8; Fig. 3) shows greater variation in iodine content of feeds with high iodine content. Of interest, all of the high iodine foods had seafood as the main flavour (Food 5, tuna; Food 6, salmon; Food 7, prawn; Food 8, haddock). The low iodine foods were flavoured beef, turkey, chicken and salmon for feeds 1, 2, 3 and 4, respectively. The coefficient of variation for iodine content between different batches of high iodine foods was from 14.5 to 31.2%, whereas for the low iodine feeds it was 13.8–26.2% (Table S3), resulting in similar overall batch-to-batch variation in iodine content (Table S3).

### Iodine content of cat urine and hair

**Figure 4:**
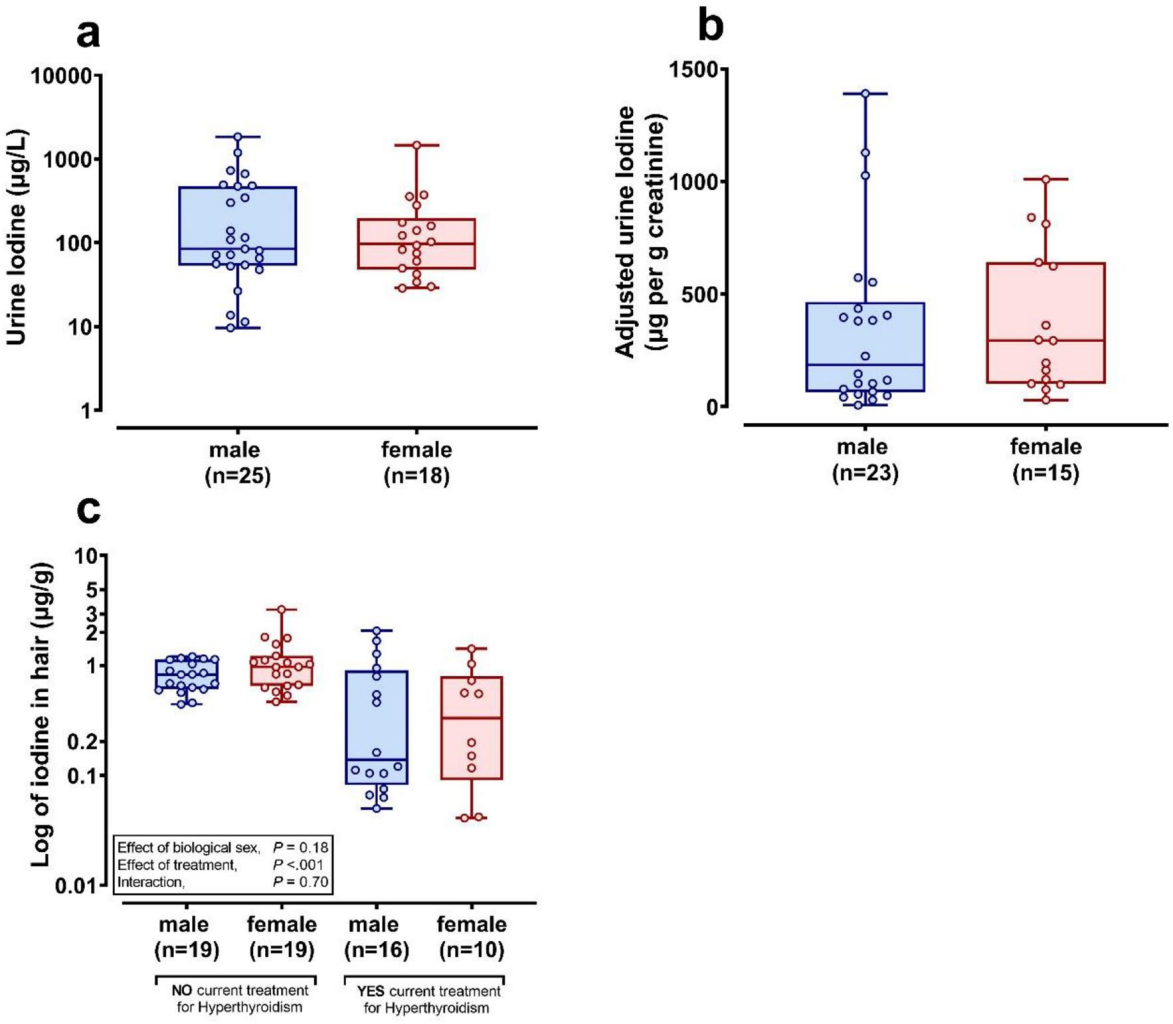
Iodine content of domestic cat urine and hair. (**a)** Iodine concentration was measured in neat urine samples (500 μl) from cats, diluted 1-in-20 with 1% TMAH using ICP-MS. (**b)** Data adjusted to urine creatinine concentration, to control for variation in urine flow. Log10-transformed data are presented as box and whisker plots on a semi-logarithmic scale **(a)**, y-axis), where boxes indicate the median, lower and upper quartiles and whiskers extend to min and max values. (**c)** Iodine in cat hair. Hair was washed consecutively with 3:1 (v/v) ethyl-ether:acetone, 5% EDTA for 1 h, then purite water (three-times), dried at 50 °C for 48 h. Washed and dried hair samples (100-200 mg) were digested with TMAH and iodine content determined in a 1% TMAH matrix using ICP-MS. Data were log10-transformed to normalise the distribution prior to statistical analysis using ANOVA for effect of sex, treatment for hyperthyroidism or their interaction. Statistical significance was accepted at P < 0.05.

Overall urine iodine was not different between male and female cats, whether adjusted or not, to urine creatinine (Fig. 4a,b), but varied between cats by orders of magnitude, suggesting variable dietary intake. For analysis of hair iodine we first assessed the methodology, in our hands, used to measure iodine in hair against the only published data in human hair^38,39^. Analysis of n = 9 human hair samples, including a human hair CRM (ERM-DB001) with known trace element quantities (e.g. Cu, 33 and Zn, 209 μg/g DM^-1^, we found that human hair had 0.364 (0.276–1.632) μg/g DM iodine, median (interquartile range), similar to that previously published 1.21 (0.098–4.21 μg/g DM^−1^) mean (range; Fig. S1c). In feline hair, the cats currently being treated for hyperthyroidism had significantly lower hair iodine content relative to the cohort not being treated for hyperthyroidism (0.156 [0.068–0.700] vs. 0.847 [0.632–1.130] μg/g DM^−1^; median [interquartile range]; *P* =  < 0.001; Fig. 4c).

## Discussion

This observational study was designed to first assess what cat owners routinely fed their cats to ascertain how variable the intake of dietary iodine may be. Second, we analysed the iodine content of a range of cat feeds. Finally, we measured iodine in cat urine and in cat hair as potential markers of variability in acute dietary iodine intake (cf. urine) and as a speculative marker of longer-term iodine exposure (cf. hair). Through a survey of cat owners, we found that the diet of cats is regularly changed on a daily-to-weekly basis, usually by the type and flavour of feed. More often than not, cats are fed either *ad-lib* or twice-a-day dry kibble, mixed with a pouch of wet food of varying flavour. Iodine content of cat feeds was broadly compliant with EU guidelines, but of the 22% that were non-compliant, the majority tended to be low in iodine and were dry kibble. Different batches of the same cat feeds had between 14 and 31% variability in iodine content. Domestic cats could therefore experience low and/or variable dietary iodine, which urinalysis of iodine confirmed; urine iodine varied by orders of magnitude. It was of interest that, reported for the first time, hair iodine content was significantly lower in cats being treated for hyperthyroidism, relative to controls. The implications of this finding warrant further investigation. The domestic cat is likely to experience variable excursions of dietary iodine that could facilitate pre-disposition to hyperthyroidism in combination with other goitrogens present in the modern, domestic environment. Such an aetiopathogenesis would mirror the development of toxic nodular goitre in humans.

### What and how are cats fed

According to our survey, which was predominantly, but not exclusively, of UK-based cat owners, nearly all were fed commercial ‘branded’ pet feed; indeed, 95.4% consumed at least half of their dietary intake as commercial food, similar to that reported previously (98.8%^40^). As fed daily, the majority of commercial feed is given as a mix (approx. 50:50) of wet and dry food, as previously described^41^. If one type of food is ever given solely, then similar to the USA^42^, it is likely to be dry kibble (24% exclusively fed dry food). In our survey, in the 2020s, it was rare for cats to only receive wet food. Interestingly, similar surveys of cat owners conducted in the 1970s reported that only 39% of cats received any commercial dry food at all. Indeed, only 2% were fed dry food exclusively^43^ (cf. 24% in our survey). By the 1980/90s, dry food was becoming more popular with > 40% cats reportedly being fed *only* commercial wet/dry food^40^. These data clearly indicate changed feeding practices of companion animals, such as the cats in our survey. A greater proportion are now fed dry food exclusively, which is likely to facilitate modern working practices. Supporting these data, a large proportion of cats in our survey were reported as being predominantly indoor, but with access to the outdoor environment. A recent survey of cat owners in America^42^ found that 82% of cats lived almost exclusively indoors. This would suggest that cats in the 2020s have a greater reliance on commercial food, supplementing their diet with prey species caught outdoors infrequently.

The type of wet foods that are fed to cats, usually mixed with dry, are most often in the form of pouches. 40% of cats received tinned foods as part of their wet food intake. Decades ago when pouches were uncommon, 85% of cats were fed wet food from tins^41,43^. Around the millennium, 30% of cat owners reported feeding tinned food, with less than 10% doing so exclusively^40^. Taken together, these results again suggest altered feeding practices over the last few decades, with a gradual drift away from feeding tinned wet food to cats. Feeding tinned foods has been linked with increased risk of hyperthyroidism in cats^6,8,9,11^, which has risen sharply over a similar time-period. Thus, although tinned, and the linings of some dry food containers, may contain goitrogenic contaminants, such as BPA and PBDEs^6,44^, we would suggest this is unlikely a direct cause of the increased rates of hyperthyroidism in cats. In contrast, fish-flavoured foods have been reported to have high iodine content, and are regularly fed to cats^32^, likely due to a belief that cats prefer fish-flavoured foods^45–47^. Nevertheless, our survey data suggested that fish-flavoured foods comprised a much smaller proportion of the cat’s diet than meat flavours. If only one flavour of cat food was fed, it was uncommon for this to be fish, but rather white meat. Despite regular (i.e. daily) changes in dietary flavour, but not in brand—the latter was rarely changed—cat owners in the UK are brand loyal and only feed fish-flavoured foods uncommonly as part of a varied, weekly change in the diet flavour.

### Pet foods have variable, but not often high, iodine content

Analysis of complete pet foods revealed wide variability in the iodine content of both cat and dog foods. To our knowledge, this is the first time that the iodine content of pet foods that are commercially available in the UK has been investigated, but the results are broadly congruous with similar studies conducted in the USA^31^, New Zealand^30^ and Germany^26^. Using recommended methods (ICP-MS with alkaline-digestion;^48,49^) we report that relatively few feeds (~ 20%) were outside of current EU guidelines^34^. Certainly, many more cat feeds were non-compliant in comparison to dog feeds, but many were close to the lower or upper nutritional guideline i.e. the cats were unlikely to be exposed to widely varying iodine content. Nevertheless, in humans, TNG occurs mostly in people with mild to moderate, not severe, iodine deficiency^18^. Therefore, if the pathogenesis of feline hyperthyroidism is akin to that of human TNG, the iodine-deficient foods that we have currently analysed *may* pose a risk for hyperthyroidism in cats *if* they were consumed over long periods of time. But, of course, currently fed foods as tested here may not necessarily reflect the iodine concentrations in foods fed during the long aetiopathogeneis of feline hyperthyroidism.

Greater variability in iodine content was found among wet, relative to dry, foods; all foods that exceeded the legal maximum were wet. Similarly, previous work had indicated that wet food tended to have greater variability in mineral content^50,51^. Nevertheless, in the current study, iodine content did not significantly vary between types of packaged wet foods (e.g. tins vs pouches, trays) and to a large extent the cats in our survey were usually fed a mix of wet and dry, or dry food only, which has a more consistent iodine content. Thus, with respect to cat foods, both iodine deficient and iodine excessive ‘complete’ diets are available. If such diets happen to be fed concurrently, it is theoretically possible for a cat to experience reasonable fluctuations in iodine intake, which, even when in the range of ‘normal’, could affect thyroid hormone levels in cats^25^. Therefore, our data does suggest that the routine feeding of cats with commercially available cat foods could inadvertently contribute toward feline hyperthyroidism through varied iodine intake. Determining causality, however, would require a suitably powered randomised, longer-term clinical trial, beyond the scope of the present study.

### Wet pet feed batch-to-batch variation in iodine content

The current, and other, studies have ordinarily only determined iodine content in a range of pet foods. To our knowledge, only one study determined iodine content in different batches—two—of the same feeds^30^. In that study, the two batches of feed were broadly similar. Here, we determined iodine content in eight different complete, wet foods for adult cats across four-to-six different batches and found an average of 22% variation between batches, particularly in those foods with higher iodine content. An individual cat fed one of these higher iodine feeds over a long period of time, may experience reasonable variation in iodine intake. Enough to underpin a TNG-like hyperthyroidism? The current study cannot answer this question but does provide the evidence for perhaps closer and repeat monitoring of thyroid levels of cats fed specific diets. Toxic nodular goitre, the form of hyperthyroidism in humans that resembles feline hyperthyroidism, is induced by excessive iodine intake after a period of iodine deficiency^16^. Nevertheless, no wet food repeatedly analysed in the current study was found to have both iodine deficient and iodine excessive batches, only reasonable variation around high or low guideline amounts^34^.

### Should we feed cats fish? Iodine intake

A previous study has reported higher iodine content of fish, relative to egg or dairy products^32^. Indeed, our pre-selected high iodine diets all had fish as the main flavour. Cat owners in our study reported feeding fish-flavoured feeds to many of the cats. However, in our hands, the iodine content of the currently analysed feeds was not significantly higher than feeds without fish as the main flavour. While some epidemiological studies have reported an association between consumption of fish-containing foods and risk of feline hyperthyroidism^8,14^, we suggest this is unlikely to be a cause-effect relationship, via iodine content. Continued analyses of further batches of feeds with fish as the main flavour would be required. Ultimately, whether any cat has consistently high or low iodine intake is better reflected through measurement of iodine excretion in urine^28,29^.

### Urine iodine is variable in cats

In the current study, domestic cats did not appear to have chronically low or high iodine intake as reflected by analysis of their urine (cf. dogs, see Figure S1b). The kidneys have a major role in maintaining iodide homeostasis by excreting > 90% of dietary iodine intake, with iodine excretion being linearly proportional to intake^28,29^, also observed in cats^26^. Furthermore, the urinary iodine concentration of healthy cats has been shown to respond to dietary iodine changes, reducing significantly following dietary iodine restriction^27^ and increasing significantly when dietary iodine is increased^25,52^. Equally, cats fed a consistent level of iodine experience little change in urinary iodine concentration^27^. Thus, urinary iodine concentrations are a useful indicator of short-term (e.g. 1–2 days) dietary iodine intake^52,53^. In this study, feline urinary iodine was highly variable, likely reflecting, therefore, highly variable dietary intake. For all the pathological specimens we had access to, unfortunately we did not have dietary histories and thus a cause-effect relationship is beyond the scope of this study. Notwithstanding the cause of death and thus reason for necropsy in the current study, the similarity of urinary iodine between cats and dogs in the current study, does not suggest that cats have very different iodine intake to dogs, at least in the short-term. Marked variability in urinary iodine within individual cats has been previously reported^54,55^. Of interest, cats with a diagnosis of hyperthyroidism but sampled prior to treatment, were found to have lower urinary iodine excretion compared to euthyroid cats^55^. In the current study, urinary iodine concentrations were measured exclusively in outwardly healthy animals. However, as a potential biomarker of longer-term iodine exposure we did sample hair in known healthy-control (free from thyroid disease) or in cats currently receiving treatment for hyperthyroidism.

### Hair iodine content is lower in cats being treated for hyperthyroidism

Since no study had previously measured iodine content of cat hair, we first validated our methods using human hair (with a human hair CRM). In our hands, human hair iodine was similar to previously published values i.e. 0.385 (0.209–0.535) μg/g iodine (median [interquartile range])^39^. No study has related hair iodine, as a longer-term marker of intake, with a diagnosis of hyperthyroidism. In cats, we report for the first time that cats being treated for hyperthyroidism had significantly lower hair iodine content than healthy cats without any known thyroid condition. Note that cats with a diagnosis of hyperthyroidism, but sampled prior to treatment, had significantly lower urinary iodine excretion compared to euthyroid cats^55^. Children with goitre have enlarged thyroid glands that correlates with higher hair iodine content^56^, which is believed to arise from chronic, excessive iodine intake^57^. However, both feline hyperthyroidism and TNG are conditions associated with an elderly population and so these data in children are of questionable relevance. Our novel data also require further validation: we were, for example, unable to determine whether hair iodine is lower in cats being treated for hyperthyroidism and thus reflect success of treatment or ‘biomarks’ a chronically low or variable-low dietary iodine status.

The study does have some limitations which should be acknowledged when interpreting the results. We were unable to retrospectively obtain diet histories for the cats submitted to pathology in our study, and thus the data for each of groups and outcomes are a cross-sectional sample, and should be treated with a degree of caution. Nevertheless, these first data do suggest that a longer-term study of euthyroid and hyperthyroid cats taking dietary, urine and hair samples is warranted to better understand the relationship between intake, uptake and bioaccumulation. Hair has been used previously as an easily obtained biomarker of longer-term mineral status, because many trace elements are permanently deposited in the hair matrix during formation^58,59^. Furthermore, other variable factors in our observational study that could be addressed in a prospectively-designed experimental study could include a possible effect of age on hair mineral analyses, as our control versus hyperthyroid cat groups were not age-matched. In addition, other comorbidities that could have an influence on iodine status but were not recorded on our database such as inflammatory bowel disease, could influence interpretation of results.

In summary, the present study has characterised estimated exposure of domestic cats to low and variable iodine intake, a dietary pattern associated with toxic nodular goitre in humans. Based on our survey data, feeding practices of domestic cats have changed considerably over the last few decades; most owners now feed commercial food, mainly dry kibble with varying types/flavours of additional wet pouches. Owners are brand loyal and tinned food is on the decline. We report good (78%) compliance of diets to European guidelines for iodine content. The 22% non-compliant feeds were mostly below nutritional minimum and were likely to be dry kibble. Feeds and urinalysis indicated a likely wide variation in dietary iodine intake in the domestic cat population. Significantly low iodine in the hair of cats being treated for hyperthyroidism may reflect success of treatment or biomark chronically low dietary intake. Labelling feeds with iodine content would empower owners to make informed decisions when selecting foods for their cats.

## Supplementary Information

Supplementary Information.
