# Supplementary material for: Estimating short and longer-term exposure of domestic cats to dietary iodine fluctuation
Source: Sci Rep. 2022 May 28;12:8987. doi: 10.1038/s41598-022-13139-8 (PMC9148307; doi:10.1038/s41598-022-13139-8)
Supplement: Supplementary file 1 — Supplementary Information. [file 41598_2022_13139_MOESM1_ESM.pdf]

Estimating short and longer-term exposure of domestic cats to dietary iodine fluctuation

Authors: Alborough, R.[1*], Graham, P[1]. and Gardner D.S.[1*]

Supplementary Information:

Table S1: The proportion of the commercial diet fed as wet food for cats in single vs. multi-cat households

Table S2: Does inclusion of all data from all cats, including those that live in the same house, affect the result compared to only using data from one cat per household?

Table S3. Variation in the iodine concentration among subsequent batches of wet cat foods (n=8) with either high or low iodine concentration.

Figure S1. Reference data for iodine in canine feed, urine and as measured in healthy human hair.

Table S1. The proportion of the commercial diet fed as wet food for cats in single vs. multi-cat households

Proportion of commercial diet that is wet food	Single cat household	Multi-cat household	Cat 1	Cat 2	Cat 3
	n=217 owners n=217 cats	n=140 owners n=318 cats	n=353 owners n=353 cats	n=136 owners n=272 cats	n=33 owners n=99 cats
**‘All’**	11 (5.06)	17 (5.35)	20 (5.67)	7 (5.19)	1 (3.03)
**‘More than half’**	43 (19.8)	68 (21.4)	76 (21.5)	27 (20.0)	5 (15.1)
**‘Half’**	72 (33.2)	84 (26.4)	105 (29.7)	39 (28.9)	9 (27.2)
**‘Less than half’**	42 (19.3)	69 (21.7)	69 (19.5)	30 (22.2)	9 (27.2)
**‘None’**	49 (22.6)	80 (25.2)	83 (23.5)	32 (23.7)	9 (27.2)
**P-value**	0.576		0.960		

Table S1. The proportion of the commercial diet fed as wet food for cats in single vs. multi-cat households A survey of cat feeding habits was created using Jisc Online Surveys and data were collected from n=362 owners of a total of n=550 cats. A multiple-choice question concerning the proportion of wet food (all, more than half, half, less than half, none) in the cat’s commercial diet was completed by n=357 respondents for n=535 cats. The data presented here are number of cats (% of total cats for that column). The Pearson chi-square test was used to determine whether the proportion of wet food in the cat’s commercial diet was different for cats that lived in multi-cat households and cats that did not live with other cats (χ^2 [4 *d.f.,* n=535] =2.89, P=0.576) and for individual cats within the same household where there were up to 3 cats (χ^2 [8 *d.f.*, n=33] =2.4, P=0.96).

Table S2. Does inclusion of all data from all cats, including those that live in the same house, affect the result compared to only using data from one cat per household?

Proportion of wet food	All cats	1st cat only	Frequency of flavour changes	All cats	1st cat only
	n=535 cats	n=353 cats		n=535 cats	n=353 cats
**‘All’**	28 (5.2)	20 (5.7)	**‘Daily’**	179 (34.2)	117 (33.6)
**‘More than half’**	111 (20.7)	76 (21.5)	**‘A few times a week’**	96 (18.3)	63 (18.1)
**‘Half’**	156 (29.0)	105 (29.7)	**‘Weekly’**	36 (6.9)	23 (6.6)
**‘Less than half’**	111 (20.7)	69 (19.5)	**‘Monthly’**	35 (6.7)	25 (7.2)
**‘None’**	129 (24.1)	83 (23.5)	**‘Less than monthly’**	60 (11.5)	44 (12.6)
			**‘Never’**	118 (22.5)	76 (21.8)

Table S2. Does inclusion of all data from all cats, including those that live in the same house, affect the result compared to only using data from one cat per household? The ‘proportion of wet food’ and the ‘frequency of flavour changes’ in the commercial diet fed to cats, when data from all cats for which the survey was completed, including those within the same household, was analysed, versus when data for just the first cat reported by each owner were analysed. The survey of cat feeding habits was created using Jisc Online Surveys. The multiple-choice questions concerning the proportion of wet food (‘All’, ‘More than half’, ‘Half’, ‘Less than half’, ‘None’) in the cat’s commercial diet and the frequency of flavour changes (‘Daily’, ‘A few times a week’, ‘Weekly’, ‘Monthly’, ‘Less than monthly’, ‘Never’) were completed by n=353 respondents for n=535 cats. The data presented here are number of cats (% of total cats for that column) when data from all cats are considered and when just the first cat reported by the owner in each survey was used.

Table S3. Variation in the iodine concentration of wet cat foods with either low (n=4) or high (n=4) iodine concentration across 4-6 different batch codes.

Food	Iodine content	Range (mg/100g DM)	Coefficient of variation, CV (%)	Average CV (%)
1	Low	0.145	26.2	16.5
2	Low	0.071	13.8	
3	Low	0.055	8.9	
4	Low	0.031	17.1	
5	High	0.573	28.8	22.4
6	High	0.282	14.9	
7	High	0.673	31.1	
8	High	0.348	14.5	

Table S3. Iodine content of feeds was measured by ICP-MS in duplicate samples (100-200mg) of freeze-dried, homogenised, TMAH-digested foods, corrected to duplicate certified reference material (CRM; SERO210705 Seronorm Trace Elements Urine L-2; LGC Standards). Between four to six subsequent batches of eight different complete, wet foods for adult cats were purchased over time.

Figure S1. Reference data for iodine in canine feed, urine and as measured in healthy human hair.

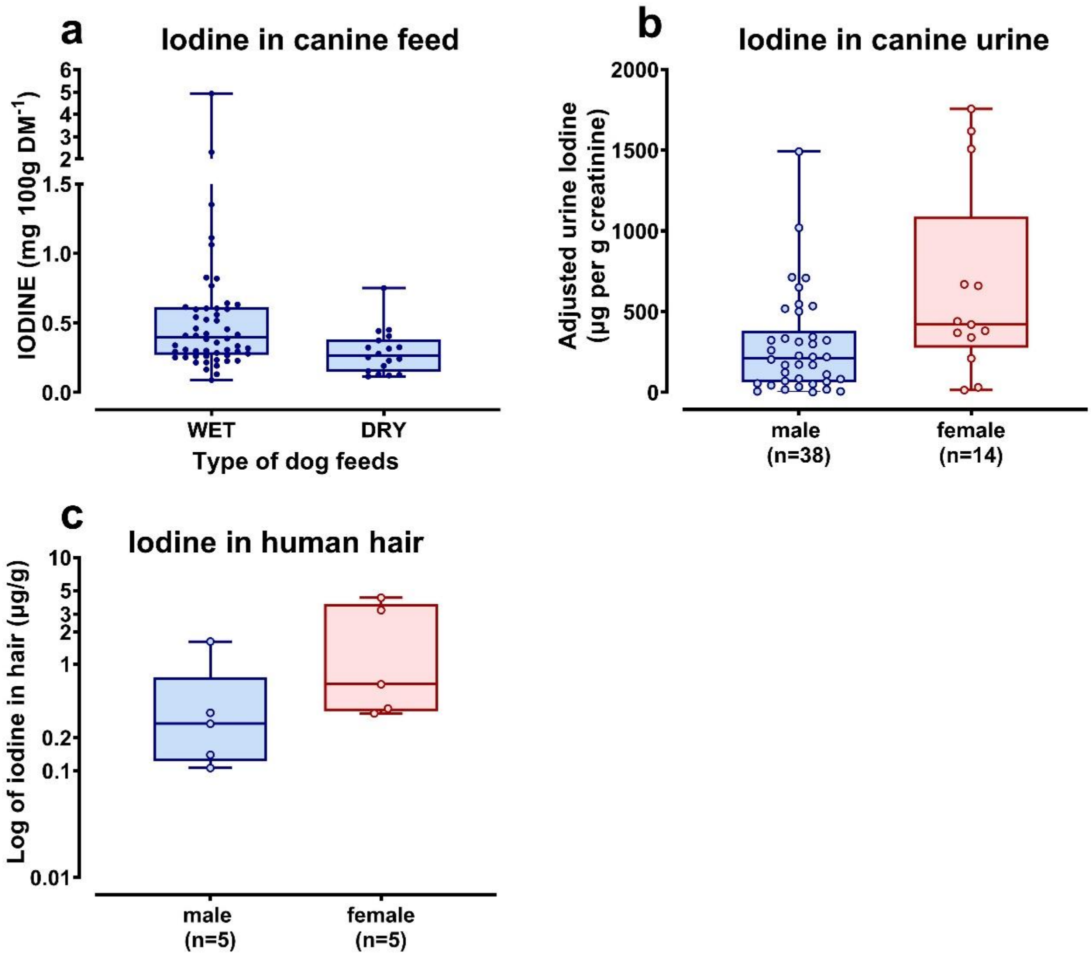

Figure. a) Freeze-dried wet and dry pet foods were homogenised and duplicate samples (100-200mg) were digested with TMAH for subsequent analysis of their iodine content by ICP-MS. The iodine content of wet and dry dog foods are each represented by an individual dot, boxes show upper to lower quartiles with line at median, whiskers from min to max. **b)** Iodine concentration was measured in neat canine urine samples (500µl), diluted 1-in-20 with 1% TMAH using ICP-MS and adjusted to the measured creatinine concentration to control for variation in urine flow. Data are presented as box and whisker plots, where boxes indicate the median, lower and upper quartiles and whiskers extend to the min and max values. **c)** since iodine in hair had only ever been reported in human hair and we had a human hair certified reference material with known trace element composition we validated the methods in our hands using human hair as collected by us in a cohort of male and female healthy volunteers (all free from known thyroid disease). Hair was washed consecutively with 3:1 (v/v) ethyl-ether:acetone, 5% EDTA for 1h, then purite water (three-times), dried at 50°C for 48 hours. Washed and dried hair samples (100-200mg) were digested with TMAH and iodine content determined in a 1% TMAH matrix using ICP-MS. Data were $\log_{10}$-transformed and are presented as box and whisker plots on a logarithmic scale. Boxes indicate the median, lower and upper quartiles and whiskers show the 10th to 90th percentiles.
